# Performance of ChatGPT in Israeli Arabic-language OBGYN national medical licensure exam

**DOI:** 10.1186/s12909-026-08753-3

**Published:** 2026-02-11

**Authors:** Adiel Cohen, Elior Eliasi, Raanan Meyer, Yoav Brezinov, Gabriel Levin

**Affiliations:** 1 Department of Obstetrics and Gynecology, Hadassah Medical Organization and Faculty of Medicine, Hebrew University of Jerusalem, Jerusalem, Israel; 2 Department of Obstetrics and Gynecology, Mayanei Hayeshua Medical Center, Sackler School of Medicine, Tel Aviv University, Bnei Brak, Israel; 3 Department of Obstetrics and Gynecology, Chaim Sheba Medical Center, Ramat-Gan, Israel; 4 Faculty of Medicine, Tel-Aviv University, Tel-Aviv, Israel; 5 Cedar-Sinai Medical Center, Los Angeles, US; 6 Lady Davis Institute for Cancer Research, Jewish General Hospital, McGill University, Montreal, QC Canada; 7 Division of Gynecologic Oncology, McGill University Health Centre, McGill University, Montreal, QC Canada; 8 Department of gynecologic oncology, Hadassah Medical Organization, Hadassah-Hebrew University Medical Center, Ein Kerem, P.O.B. 12000, 91120 Jerusalem, Israel

**Keywords:** Arabic, ChatGPT, Medical exam, OBGYN, Performance

## Abstract

**Background:**

Previous studies of ChatGPT performance in the field of medical exams have reached contradictory results. The performance of ChatGPT in languages other than English, including Arabic, which is the official language of medical education and practice in many countries, has yet to be explored. We aim to evaluate the performance of ChatGPT in Arabic-language Israeli OBGYN medical licensure exams for foreign university alumni.

**Methods:**

We conducted a performance study using a consecutive sample of text-based multiple-choice questions, originated from authentic Arabic-language Israeli OBGYN medical licensure exams for foreign university alumni. ChatGPT-3.5 (using a newly created account) answered all questions in Arabic. We compared the performance of ChatGPT including in the different fields of the exam; Obstetrics, Reproductive medicine and Infertility, Gynecology and Gynecologic Oncology, and also compared ChatGPT Arabic performance vs. previously published English medical tests.

**Results:**

Overall, 123 authentic questions were analyzed. ChatGPT correctly answered 54 questions (43.9%, 95% CI: 35.1% – 52.7%) and reached a score below 50%. There was no difference in ChatGPT performance in the four different subjects of the exam: Gynecologic Oncology (61.5%, 95% CI: 35.1% – 87.9%), Gynecology (44.0%, 95% CI: 24.5% – 63.5%), Obstetrics (42.3%, 95% CI: 28.9% – 55.7%), Reproductive medicine and Infertility (39.4%, 95% CI: 22.7% – 56.1%), *p* = .579. In a comparison to ChatGPT performance in 9,091 English language questions in the field of medicine, the performance of Arabic ChatGPT was lower (43.9% in Arabic vs. 60.7% in English, *p* < .001).

**Conclusions:**

ChatGPT-3.5 answered correctly approximately 44% of Arabic OBGYN medical licensure exam questions. At the time of writing of this manuscript, considering the results of our analysis, ChatGPT-3.5 cannot be considered a reliable primary tool for exam preparation in Arabic. Further research and efforts should be made to improve ChatGPT performance in other languages besides English especially Arabic.

## Background

Full medical registration in Israel requires passing medical state examinations, taken during the last two years of medical school, and passing an internship period of one year [1]. One of the professions in the examination is obstetrics and gynecology (OBGYN). As acceptance to Israeli medical schools is highly competitive [2], many young people choose to undergo their academic medical training abroad [3]. Although these studies are recognized in Israel, their graduates are still required to successfully pass a local licensing test. For these alumni, the licensure test is different from the locally trained students, and it can be taken in a few languages, one of those is Arabic. This licensure exam consists of 30 multiple choice questions in OBGYN.

Traditional methods of preparation for these exams include memorizing textbooks, participating in special directed courses and practicing through available multiple-choice questions in a period of several months of preparation. However, with the advent of artificial intelligence (AI), it is becoming increasingly common to use digital tools to aid in exam preparation [4–6]. Chat Generative Pre-trained Transformer (ChatGPT), at the time this manuscript was written was almost a year old powerful machine learning-based language model, has overwhelming performance in natural language processing and is currently revolutionizing the field of medicine [7–10].

Previous studies of ChatGPT performance in the field of medical exams have reached contradicting results [6, 11, 12].

Nevertheless, the performance of ChatGPT in languages other than English, specifically Arabic, which is the official language of medical education and practice in many countries [13], has yet to be explored. This study aims to evaluate the performance of ChatGPT in Arabic-language Israeli OBGYN medical licensure exams for foreign university alumni.

## Methods

### Medical test data sets

#### Medical licensure exam

In Israel, under the 1976 Physicians’ Ordinance, the Ministry of Health is responsible for granting medical licensure [1]. After completing 6 years of medical school, including 5 medical licensure exams taken during the last two years of medical school: OBGYN, pediatrics, internal medicine, surgery, and psychiatry—graduates must complete a one-year internship, following which they receive a medical diploma. For medical students trained abroad, which now constitutes more than 50% of newly licensed Israel Medical Doctors (MD), there is a specific exam. The goal of this exam is to evaluate the level of knowledge and knowledge application of the students. To identify the students whose level of knowledge and application of knowledge does not reach the required minimum and to create a uniform assessment level, at the requested standard. This licensure exam takes place twice a year and is composed of 210 questions in the previously mentioned five fields of medicine. The OBGYN section consists of 30 questions in a multiple-choice format. The passing grade is 60 (calculated as correct answered divided by 210) and the exam can be taken in the following languages: Hebrew, English, Arabic, Spanish, French and Russian.

For the purpose of this study, we accessed the authentic OBGYN sections of the published exams (2021 and on) and their correct solutions. We included all eligible exams in this period. We consecutively included text-only questions (excluding pictures and figures-based questions). To ensure an unbiased conversation history, a new ChatGPT-3.5, free interface account was created specifically for this study. The questions were formatted exactly as they appeared in the question stem. For each question, the following prompt was provided to ChatGPT: « يرجى قراءة السؤال التالي واختيار الإجابة الصحيحة من الإجابات المحتملة» English translation: “Please read the following question and select the correct answer from the possible answers»). To avoid any influence of conversational context on model outputs, each question was entered into a separate new ChatGPT session, ensuring that the model had no memory of prior prompts. Although ChatGPT does not update its underlying training or parameters during user interactions, its responses can be influenced by conversation history within an active chat. Opening a new session for each item prevented such contextual carry-over and ensured standardized, independent responses. *English.*

#### Language performance of ChatGPT

To contextualize Arabic-language performance, we sought to compare it to English language performance at time the study was performed. We have searched PubMed for all publications with the term “ChatGPT”. Out of 502 publications (search date 28th May 2023) we conducted a manual review of each title and abstract, in cases where abstracts were unavailable, we thoroughly examined the full texts. Our search focused on manuscripts evaluating the performance of ChatGPT in answering medical-related questions. We specifically included publications that evaluated ChatGPT's performance in a binary manner (right/wrong) and excluded publications that assessed and graded/scored ChatGPT's answers. Because these studies may vary markedly in design (specialty, exam type, question structure, and ChatGPT version), we used a simple descriptive approach. When question-level counts were available, we calculated a question-weighted accuracy (total correct responses divided by total questions). When a study did not report the number of questions, we included it only in the narrative comparison and excluded it from the quantitative pooled estimate. This approach does not constitute a meta-analysis and does not account for between-study heterogeneity or publication bias; therefore, the pooled proportion should be interpreted as a broad descriptive benchmark rather than an inferential comparison. Overall, 16 studies were included in the final analysis [11, 12, 14–27].

#### Data analysis

All answers were recorded into a single database. Two Reviewers (AC and GL, a resident in OBGYN and a specialist in OBGYN and subspecialist in Gynecologic Oncology) manually reviewed each question. We reviewed each question and defined the subject of the question based on the content and context of the question. The categories were as follows: Obstetrics, Gynecology, Gynecological Oncology, and Reproductive medicine and Infertility. There was no disagreement between categorize among both Reviewers. Duplicates were identified manually by visual inspection of the text of the question.

#### Statistical analysis

The primary outcome was performance of ChatGPT in the Arabic-language OBGYN licensure exam (proportion of right answers/all answers). Our secondary measure was to compare ChatGPT performance in Arabic-language vs. ChatGPT performance in English language as published in known literature. We used descriptive statistics to compare ChatGPT performance in the different sections of the test and to compare the performance of the ChatGPT Arabic-language test to its published performance in English language. We performed Chi-square test to compare proportions of right answers in English and Arabic. For all statistical analyses, a two-sided *P* < 0.05 was used as the criterion for statistical significance. All analyses were conducted using SPSS 29 (SPSS Inc., Chicago, IL).

#### Ethical approval

We used American Association for Public Opinion Research (AAPOR) reporting guidelines. This study did not require ethics approval, as we used only publicly accessible data, and no human participants were involved. No access to confidential or unpublished examination material occurred.

## Results

Overall, 150 authentic questions were evaluated (six exams, of which one was published twice). There were 27 (18.0%) questions involving figures and pictures, resulting in 123 questions available for analysis. Fifty-two (42.3%) questions in Obstetrics, 33 (26.8%) in Reproductive medicine and Infertility, 25 (20.3%) in Gynecology and 13 (10.6%) in Gynecologic Oncology (Table 1).

**Table 1:** Characteristics of the test performance of ChatGPT in Arabic-language

Characteristic	n (%)	*p* value
Composition of exam parts		
Field		
Obstetrics	52 (42.3%)	
Reproductive medicine and Infertility	33 (26.8%)	
Gynecology	25 (20.3%)	
Gynecological Oncology	13 (10.6%)	
Performance of ChatGPT in various parts of the exam*		
Field		.579
Gynecologic Oncology	8/13 (61.5%)	
Gynecology	11/25 (44.0%)	
Obstetrics	22/52 (42.3%)	
Reproductive medicine and Infertility	13/33 (39.4%)	

Numbers are described as numbers (proportion)

^*^Number of correct answers

### Arabic test performance

The proportions of the different parts of the test are described in Table 1. ChatGPT correctly answered 54 questions (43.9%, 95% CI: 35.1% – 52.7%).

There was no difference in ChatGPT performance in the four different subjects of the exam: Gynecologic Oncology (61.5%, 95% CI: 35.1% – 87.9%), Gynecology (44.0%, 95% CI: 24.5% – 63.5%), Obstetrics (42.3%, 95% CI: 28.9% – 55.7%), Reproductive medicine and Infertility (39.4%, 95% CI: 22.7% – 56.1%), *p* = 0.579.

### Arabic-language vs. English language ChatGPT performance

In a comparison to ChatGPT performance in English language in the medical field, in 16 published studies (total 9,091 questions) pooled descriptive accuracy of ChatGPT in English was higher 60.7% (95% CI: 59.7% – 61.7%) when compared to our Arabic-language dataset test 43.9% (95% CI: 35.1% – 52.7%) *p* < 0.001 (Table 2).

**Table 2:** Studies describing ChatGPT performance in medical exams in English language

Study	Number of questions	Performance (%)	Field
Humar et al. [14]	1,129	55.8%	Plastic surgery
Gupta et al. [15]	242	54.9%	Plastic surgery
Mihalache et al. [16]	125	46%	Ophthalmology
Giannos et al.[17]	126	40%	Biomedical admission test
Nakhleh et al.[18]	24	100%	Diabetes
Subramani et al. [19]	20	85%	Medical physiology
Hopkins et al.[20]	643	53.2%	Neurosurgery
Kung et al.[12]	350	60%	USMLE
Fijacko et al. [21]	126	69.1%	ACLS
Gilson et al. [11]	389	52.0%	USMLE
Huh et al.[22]	79	60.8%	Parasitology
Lum et al. [23]	207	47%	Orthopedics
Suchman et al.[24]	455	65.1%	Gastroenterology
Birkett et al.[25]	3,705	69.7%	Anesthesia
Shay et al.[26]	1,321	56.2%	Anesthesia
Bhayana et al.[27]	150	69%	Radiology
**Total**	**9,091**	**60.7%**	

*USMLE* United States Medical Licensing Examination, *ACLS* Advanced Cardiovascular Life Support

Performance is number of right answers/number of answers

## Discussion

Our study found that ChatGPT-3.5 in Arabic language—correctly answered less than 50% of the Israeli medical licensure exam in OBGYN, and there was no varying performance across different parts of the OBGYN test. ChatGPT-3.5 in Arabic reached lower scores than published in studies in English language, though interpretation is limited due to heterogeneity in question types.

As ChatGPT's involvement in medical education continues to advance, it is essential to underscore the responsible use of this tool. Although there is a growing body of literature regarding ChatGPT's performance in the medical field, there is a notable gap in its practical use for medical education in languages other than English, notably Arabic.

ChatGPT has been extensively trained on an extensive corpus of English text, leading to its remarkable proficiency across a range of natural language processing tasks, encompassing language generation and comprehension [28]. Abundant evidence demonstrates ChatGPT’s exceptional ability to generate coherent and contextually appropriate responses for a diverse array of prompts, ranging from casual conversations to complex inquiries. Notably, discerning between medical texts authored by ChatGPT and those written by humans is exceedingly challenging [29, 30].

ChatGPT's significant contribution to the field of medicine lies in its ability to understand and generate meaningful responses to natural language inputs [31].

At the time the study was conducted, ChatGPT demonstrated impressive performance in Arabic within the scope of this study. Because our study did not directly evaluate the size or nature of ChatGPT’s training corpora, any assumptions regarding the specific causes of performance variability should be interpreted cautiously. Future model iterations may address these limitations, particularly if developers incorporate more diverse and comprehensive Arabic-language data; however, such improvements remain speculative and were not assessed in this analysis.

Interestingly, although not statistically significant, higher absolute scores were notable in Gynecologic Oncology. Interpretation of statistically non significance numerical difference should be done with extreme cautious, specifically when sample size is the limiting factor. This finding is difficult to explain and perhaps should be further evaluated in future research.

Our study underscored the necessity for Arabic-speaking medical students to exercise caution, attentiveness, and a heightened level of scrutiny when using ChatGPT in preparation for exams. We discovered that the tool's efficacy in aiding preparation for medical exams in the Arabic language falls short in comparison to its performance in English.

This is the first report of which we are aware, testing the ability of the ChatGPT in Arabic language. Nevertheless, our study has some limitations. Our sample size is relatively small, single-country dataset. As the study is restricted to a specific context – the generalizability is limited to it’s context and may be hypothesis generating with pertains to other fields. ChatGPT offers distinct responses, and replicating our study using the same methodology may lead to varying outcomes. We did not examine the performance of ChatGPT in handling questions embedded within figures and tables, nor in responding to open-ended queries. It is important to avoid generalizing our findings to other languages, as it is probable that ChatGPT will swiftly enhance its knowledge in various languages, including Arabic. Lastly, our comparison with English-language performance relies on aggregated published data with substantial heterogeneity; thus, the pooled English estimate should be regarded as descriptive rather than causal, and cross-language conclusions should be interpreted cautiously.

## Conclusion

In conclusion, in the Arabic-language OBGYN medical licensure exam, ChatGPT-3.5 achieved an accuracy rate of approximately 44% in answering questions correctly, lower than it’s performance in English medical examinations. Arabic speaking Israeli student should acknowledge the progress of AI in the field of medicine. However, it's crucial to recognize that, as indicated in this study, at the time of writing of this manuscript, considering the results of our analysis, ChatGPT-3.5 cannot be considered a reliable primary tool for exam preparation in Arabic. Further research and efforts should be made to improve ChatGPT performance in other languages besides English especially Arabic.

## Data Availability

Publicly available exam content was used. The datasets generated during and analyzed during the current study are not publicly available due to confidentiality reasons but are available from the corresponding author on reasonable request.

## Abbreviations

OBGYN: Obstetrics and gynecology
ChatGPT: Chat Generative Pre-trained Transformer
